# Results of Neuroimaging in Patients with Atypical Normal-Tension Glaucoma

**DOI:** 10.1155/2020/9093206

**Published:** 2020-08-18

**Authors:** Ewa Kosior-Jarecka, Dominika Wróbel-Dudzińska, Radosław Pietura, Anna Pankowska, Beata Szczuka, Iwona Żarnowska, Urszula Łukasik, Tomasz Żarnowski

**Affiliations:** ^1^Department of Diagnostics and Microsurgery of Glaucoma, Medical University of Lublin, Lublin, Poland; ^2^Department of Radiography, Medical University of Lublin, Lublin, Poland; ^3^Department of Paediatric Neurology, Medical University of Lublin, Lublin, Poland

## Abstract

**Aim:**

The aim of the study was to determine the frequency of pathologies which can mimic normal-tension glaucoma (NTG), observed in neuroimaging of NTG patients, and to evaluate the frequency of pathologies in determined additional indications for neuroimaging. *Material and Methods*. The studied group consisted of 126 NTG patients who met at least one of the following criteria: unilateral NTG, damage in the visual field (VF) inconsistent with optic disc appearance, fast VF progression, worsening of visual acuity, predominant optic disc pallor rather than optic disc excavation, diagnosis under the age of 50, and scotoma in VF restricted by a vertical line. The patients included in the research underwent MRI scans of the brain and both orbits.

**Results:**

After neuroimaging, the results of 29 (23%) patients were qualified as positive; 18 (14.2%) of the identified pathologies were found to clinically affect the visual pathway. The most frequent brain pathology was intracranial meningiomas, observed in 4 patients (3.1%), followed by optic nerve sheath meningiomas diagnosed in 3 cases (2.4%), and brain glioma in 1 patient (0.8%). Pituitary gland adenomas were described in 6 patients (4.5%); 3 of the tumours were in contact with the optic chiasm. 53 (40%) patients had minimal ischemic changes in different regions of the brain. In the case of worsening BCVA or fast VF progression, the frequency of positive results was the highest (50% and 40%), whereas in the case of diagnosis at a young age and unilateral involvement, neuropathology was the rarest (0% and 6.9%).

**Conclusions:**

In the case of NTG, the decision to perform neuroimaging should be made after a detailed assessment of clinical status, rather in the event of finding the signs of possible compressive optic neuropathy than as an obligatory procedure for every patient.

## 1. Introduction

Normal-tension glaucoma (NTG) is a form of primary open-angle glaucoma with intraocular pressure (IOP) never exceeding 21 mmHg. In this form of the disease, IOP may not be the main causative factor and different theories explaining the pathogenesis are postulated [1–6].

In the case of NTG, detailed differential diagnostics should be performed before establishing the diagnosis because optic disc cupping is not pathognomonic for glaucoma. Glaucoma with previously or periodically increased IOP needs to be excluded (pigmentary glaucoma and steroid or inflammatory glaucoma, also the cases with wide daily fluctuations in IOP). Excavated disc may be observed in the course of ischemic neuropathy, inherited anomalies, and compressive neuropathy [7]. The distinction between NTG and the last of these conditions is crucial because it excludes underlying life-threatening conditions.

The differential diagnosis procedure for distinguishing between NTG and compressive neuropathy of the optic nerve is neuroimaging. However, the indications are not clear. Some ophthalmologists point to the need to give an MRI scan to every NTG patient, while others perform neuroimaging only in the presence of specific additional clinical features (in addition to IOP within the normal range) that are not typical for glaucoma [8, 9].

The aim of the study was to determine the frequency of pathologies which can mimic normal-tension glaucoma, observed in the neuroimaging of NTG patients, and to evaluate the frequency of pathologies in each of the additional indications for neuroimaging.

## 2. Material and Methods

The study was designed as a prospective one-centre study in the Department of Diagnostics and Microsurgery of Glaucoma in the Medical University of Lublin (Poland) in 2014-2017. The study design was accepted by the local ethics committee and conformed to the standards set by the Declaration of Helsinki. The study was funded thanks to the clinical grant of the Medical University of Lublin. All patients who were diagnosed with normal-tension glaucoma, met the inclusion criteria, and consented to their participation were included in the study. Patients were diagnosed with normal-tension glaucoma according to the following criteria: glaucomatous neuroretinal rim loss in at least one eye, open angle in gonioscopy, and intraocular pressure (IOP) consistently <21 mmHg (with the highest value ever measured in a given patient being ≤21 mmHg), and no detectable eye pathology which would imply a diagnosis of secondary glaucoma (neovascularisation, pseudoexfoliation, or pigment dispersion syndrome). Before the diagnosis, the maximal IOP was checked during 2 separate visits during different time points and during every visit, it was measured twice with a 2-hour break. Single time-point IOP measurement consisted of 3-time IOP check by a single observer, and the mean value was counted. The calibration of an applanation tonometer was performed routinely at the beginning of the office hours. Only the patients with IOP never exceeding 21 mmHg (corrected according to central corneal thickness values) were included. In the course of diagnosis, the Humphrey visual field test 30-2 was performed on all patients with BCVA better than 0.05, as well as an assessment of the morphology of scotoma. The VF results were evaluated, and the diagnosis was confirmed by glaucoma specialists (EKJ, UL, or TZ). Glaucoma was defined based upon the clinical determination of glaucomatous ONH damage (localized or diffuse neuroretinal rim thinning, rim notching, excavation, and/or RNFL defect) associated with typical, reproducible standard automated perimetry (SAP) defects. Glaucomatous defect on SAP was defined based upon a glaucoma hemifield test result outside normal limits and the presence of at least 3 contiguous test points within the same hemifield on the pattern deviation plot at *P* < 1%, with at least 1 point at *P* < 0.5%, on at least 2 consecutive tests, with reliability indices better than 15%. The progression rate was calculated according to clinical method by counting the difference between MD indicators from 2 reliable visual fields from the time of the diagnosis and the time of inclusion to the study. Then, this difference was calculated for one year of observation, and in case the result was higher than 1.5 dB/year, the patient was included.

The inclusion criterion was at least one of the following:

1. Unilateral normal-tension glaucoma
2. The damage in the visual field inconsistent with optic disc appearance
3. Fast visual field progression (1.5 dB per year or more) assessed according to at least 3 reliable visual field results
4. Worsening of visual acuity of at least 2 lines in the Snellen chart connected neither with lens nor retinal pathology
5. Optic disc excavation accompanied by pallor
6. Patients diagnosed under the age of 50
7. Scotoma restricted by a vertical line (hemianopia, quadrantanopia, and bitemporal defect).

Unilateral glaucoma as an inclusion criterion was diagnosed when during the inclusion visit, the fellow eye has no detectable features of glaucoma, neither observed in the ophthalmoscopy, nor VF examination, nor RNFL thickness.

All patients meeting the inclusion criteria who agreed to participate in the research had MRI scans performed on the brain and both orbits with a 1.5-Tesla Magnetic Resonance Scanner (Optima 360, GE Healthcare). The scans were carefully evaluated by experienced neuroradiologists (RP, BS). Any pathology found was classified as either possibly interfering with the glaucoma diagnosis or not significant.

Routine brain scans were obtained in 3 planes (axial, coronal, and sagittal) before and after intravenous administration of a paramagnetic contrast agent. Fast spin echo, diffusion weighted imaging, and fluid attenuated inversion recovery sequences were obtained producing T1- and T2-weighted images.

To evaluate the orbits, two-plane images (axial and coronal) were acquired. Fast spin echo, short time inversion recovery, and T1- and T2-weighted sequences with fat saturation before and after intravenous administration of a paramagnetic contrast agent were obtained. Acquisition parameters of the orbit examination are listed in Table 1.

Statistical analysis was performed using Statistica 13 software (StatSoft, Poland), and *P* level less than 0.05 was regarded as statistically significant.

## 3. Results

The group of NTG patients included in the studied group consisted of 126 persons: 78 (61.9%) female and 48 (38.1%) male. The mean age of patients was 66.7 years (ranging from 42 to 90 years). The demographic and clinical characteristics of the studied group are put in Table 2.

After neuroimaging, the results of 29 (23%) patients were qualified as pathological; 18 (14.2%) of the identified pathologies were found to clinically affect the visual pathway either by compression or mass effect.

Among the pathological results, the most frequent brain pathologies were intracranial meningiomas observed in 4 patients (3.1%), followed by optic nerve sheath meningiomas diagnosed in 3 cases (2.4%) and brain glioma in 1 patient (0.8%).

A considerable group of pathologies were located in the pituitary gland, with pituitary gland adenomas described in 6 patients (4.5%). Three of the tumours were in contact with the optic chiasm, and in 3 cases, there were no signs of compression. Additionally, 1 patient was diagnosed with a Rathke cleft cyst (0.8%) causing deformation of the optic chiasm and optic nerves. Seven patients were diagnosed with empty sella (4.8%)

**Figure 1:**
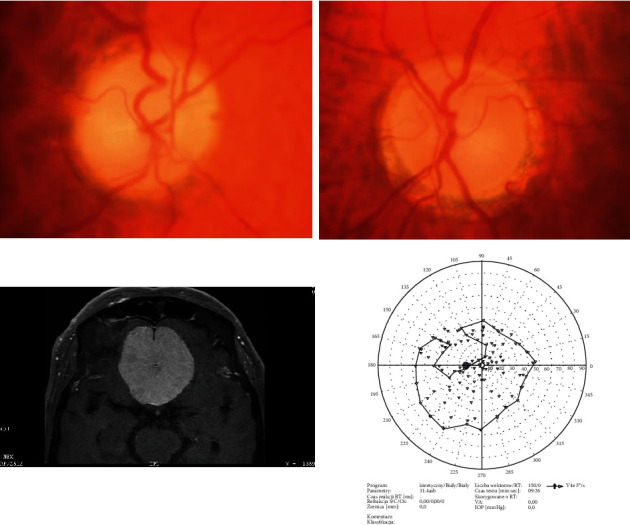
M/68 with positive history of glaucoma. The reason for neuroimaging was the decrease in BCVA from BE: RE from 0.8 to no light perception and LE from 0.8 to counting fingers up to 1.5 m with IOP at the level of 8-10 mmHg. MRI scans showed intracranial meningioma with the diameter 5.5 cm.

**Figure 2:**
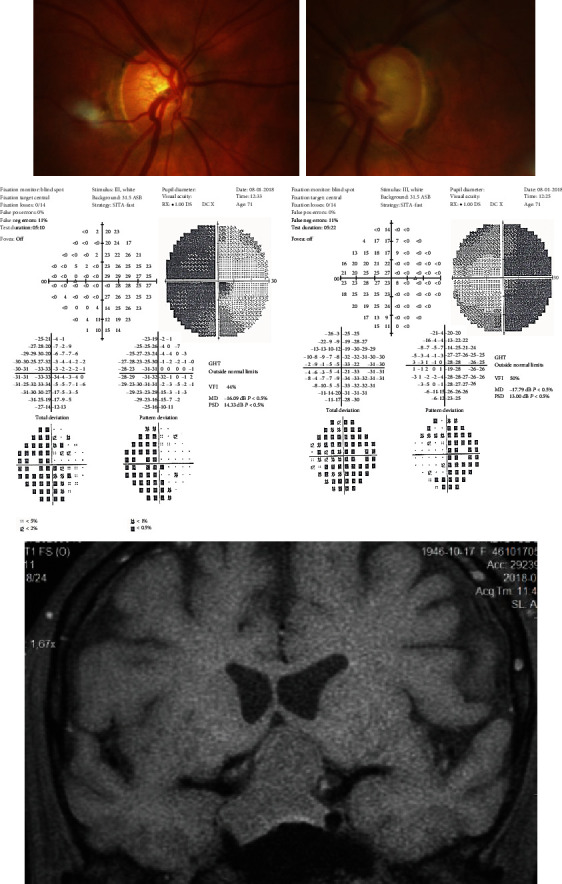
F/68 referred with glaucoma progression, neuroimaged because of bitemporal hemianopia. MRI scans revealed pituitary adenoma compressing and elevating optical chiasm.

**Figure 3:**
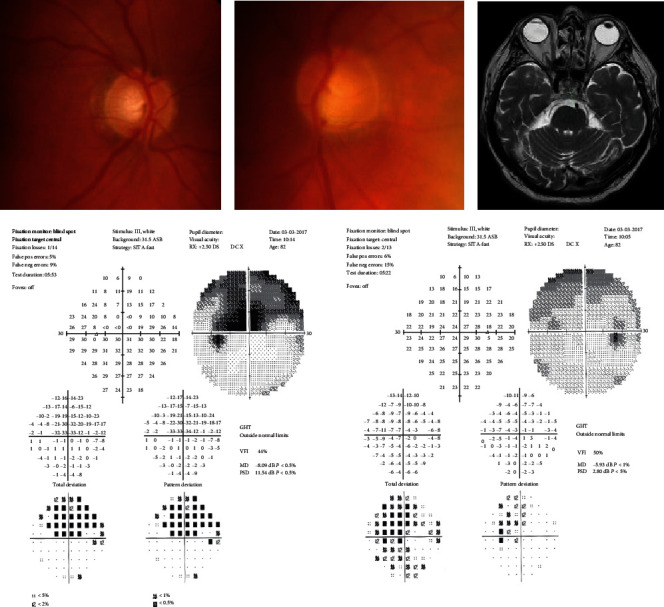
M/82 neuroimaging was performed because of unilateral NTG. MRI scanning revealed 4 mm hypophyseal adenoma which did not compress the visual pathway.

In one (0.8%) patient imaged because of unilateral glaucoma, an aneurysm in the anterior communicating artery was described which did not compress the anterior visual pathway; and the MRI examination of 1 (0.8%) patient revealed vein malformation clinically insignificant for visual pathways. One patient (0.8%) had carotid cavernous fistula with flow voids. Four (3.1%) patients showed brain changes typical of a recent stroke. The exemplary cases of NTG patients from the studied group are shown in Figures 1–3.

53 (40%) patients had minimal ischemic changes in different regions of the brain.

The data used to support the findings of this study are available from the corresponding author upon request.

In Table 3, the number of patients diagnosed because of additional indications is shown with the percentage of clinically significant results.

The total sum exceeds 100% as there were patients diagnosed for 2 reasons, mainly reasons 1 and 6 together.

In unilateral NTG, the mean MD in patients with positive MRI results did not differ significantly from patients with negative results (-10.45 dB vs. -8.08 dB; *P* = 0.1101).

## 4. Discussion

The most common cause of excavation of the optic disc is glaucoma. However, up to 20% cases may result from other pathologies, with the compressive neuropathy as clinically most relevant; in this case, early diagnosis is vision- and even life-saving [10]. As previously shown, the risk of another pathology underlying excavation is minimal in high-tension glaucoma [11]. The patients with an excavated disc in the absence of elevated IOP as the main causative factor of glaucoma are the most at risk of being misdiagnosed. There is a debate among glaucoma practitioners about the indications for neuroimaging in NTG patients [8, 9]. Some claim that such examination is obligatory in differential diagnosis because of the risk of overlooking life-threatening pathologies. However, this time- and money-consuming strategy is not always rational or even possible in busy hospitals. We decided to scan patients fulfilling at least one additional inclusion criterion which makes NTG, an atypical form of primary open-angle glaucoma, even more peculiar. The inclusion criteria in this study were established after reviewing the literature.

In the current study, the most common reason for neuroimaging in NTG patients was unilateral involvement. NTG is believed to be dependent on general rather than ocular factors, and that is why it is supposed to be more symmetrical than POAG with high initial IOP. However, in our group, only 6.9% patients with unilateral excavation had clinically significant pathology in the brain. It was the penultimate reason for neuroimaging as far as the frequency of found pathologies is concerned. It may indicate that asymmetry is quite typical at least in some NTG cases: about 25% patients with NTG presented with unilateral field loss at diagnosis [11]. Additionally, patients with NTG may present with asymmetric field loss [12] related to unequal IOP [13, 14] or ocular blood flow [15] between the eyes.

As unilateral NTG was the most frequent indication for neuroimaging in our study, we looked into the clinical records of the patients with clinically significant results trying to find similarities. In some cases, the high discrepancy between the VF of the healthy and the affected eye was obvious, making it a possible criterion for further diagnostics. Additionally, the detailed evaluation of VF pattern showed that the morphology of these scotomas was not typical in the course of glaucoma. However, an almost similar group of patients, mainly with pituitary gland tumours, had no scotoma in the affected eye.

The mean age of NTG patients reported in many studies is around 60 [16]; in another study by the authors of this article, the patients' mean age was about 70 [17], which is similar to the mean age of the participants of the current study. Younger age (under 50) at diagnosis in the current study was never related to clinically significant pathologies detected at neuroimaging. It seems that prevalence of NTG in younger patients, just like unilateral involvement, may be a feature of the typical course of NTG.

Compressive neuropathy typically presents with pale optic disc and much less frequently with excavated pale disc; both cooccur with the clinical features of optic nerve atrophy. Classical clinical findings of compressive neuropathy are slowly progressive visual loss and optic nerve atrophy. Some clinicians believe it is possible to distinguish between excavation of the optic disc caused by glaucoma and compressive lesion of the anterior visual pathways only on the basis of clinical features. The possibility of glaucoma specialists successfully distinguishing between glaucomatous and nonglaucomatous neuropathy on the basis of colour fundus photography and VF results was evaluated in some studies, and up to 88.1% of glaucoma cases and 75% of optic neuropathy cases were correctly classified [18]. Other studies reported an accuracy of 75–80% in diagnosing glaucoma and an accuracy below 50% in diagnosing other optic neuropathies [10, 19]. Greenfield et al. [20] concluded that compressive lesions of the anterior visual pathway should be evident on clinical examination, including more specific features: optic nerve pallor, vertically aligned visual field defects, and visual acuity less than 20/40. Our study confirmed that vertical VF defect was related to brain pathology in 71.4% of cases, to worsening BCVA in 50% of cases, and to pale disc in 18.1%. NTG suspect patients with these symptoms clearly need neuroimaging.

The mean progression rate in NTG is 0.75 dB per year [16], and VF is believed to be stable in NTG in the majority of cases [21]. Rapid progression, more than 1.5 dB per year, may indicate underlying compressive neuropathy, which was the case in 40% of patients scanned for this reason. Therefore, a detailed analysis of pattern defect and progression rate seems to be crucial in deciding who should be MRI scanned.

In the case of 18 patients (14.2%), the results of neuroimaging revealed a pathology which affected the anterior visual pathway, with intracranial meningiomas as the most frequent. Kalenak et al. [22] reported a patient with disc excavation and visual field loss which occurred in the course of intracranial meningioma. Shiose et al. [23] showed that 5.7% (8/141) glaucoma suspects diagnosed by optic disc examination were found to have intracranial lesions. In the current study, clinical picture of NTG in 4 patients was associated with advanced intracranial meningioma. Three of these patients had positive family history for glaucoma and had been treated with antiglaucoma medications for many years. The reason for neuroimaging was a quick decrease in VA typical of optic neuropathies other than glaucoma. The neurosurgical intervention managed to improve VA in 2 cases with the shift observed in OCT pattern from nonspecific to typical for glaucoma. It is unclear whether the mechanism of NTG development in these patients was a direct result of optic nerve susceptibility to intraocular pressure within the normal range or/and the compression itself. Additionally, it points the possibility that in some cases, compressive neuropathy accompanies NTG, which raises the question of the need for treatment decreasing intraocular pressure in patients with disc excavation and confirmed compression in anterior visual pathways.

The frequency of pathology affecting visual pathways observed in this study confirmed the reports by Stewart and Reid (8%; 2/25) [24] and Stroman et al. (15%; 3/20) [25]. Both cited studies included all patients diagnosed with NTG, whereas our study introduced an additional criterion. However, neuroimaging in every case of NTG seems to be as sensitive as only scanning the patients with this additional criterion, which confirms the results of the study by Greenfield et al. [20], who concluded that compressive lesions of the anterior visual pathway are uncommon and suggested that routine neuroradiologic screening of NTG patients is of little clinical value.

In this study, 38% of diagnosed pathologies did not affect the anterior visual pathway and were not the cause of disc cupping. Most of the participants of the research were patients diagnosed with primary empty sella and patients with pituitary microadenoma. Beattie and Trope [19] had previously reported that the coexistence of primary empty sella and glaucomatous optic neuropathy is likely a coincidence. In the case of positive MRI results, it is crucial to assess the influence of the brain pathology on the clinical picture of NTG before the diagnosis of compressive optic neuropathy is established.

The most prominent and common disturbances described in our group of NTG patients were ischemic foci located in different regions of the brain. As the group was not age-matched, we cannot distinguish between a pathology related to NTG and the symptoms of normal aging. However, there are data reporting that ischemic changes in the brain are more common in NTG patients than in control subjects, with the frequency up to 40% [25–28], which suggests that vascular insufficiency in the central nervous system has some relation to the pathogenesis of NTG. Harris et al. [29] reported reduced cerebrovascular blood flow velocity and vasoreactivity in open-angle glaucoma patients compared to age-matched control subjects. Additionally, according to some authors, brain ischemic lesions may be associated to some extent with the pattern of visual field damage [28] and progression [30] in NTG patients.

The study has some potential limitations. First, it was conducted in one centre and the results may be different in different populations. Additionally, presence of relative afferent pupillary defect which may be helpful in indication was not included as inclusion criterion. The authors decided to include patients with clinically unilateral form of the disease. The follow-up of the included patients was not planned. That is why there is the possibility of misdiagnosing the patients with asymmetric NTG at presentation as unilateral disease. All patients from the group of young NTG patients were negative in MRI neuroimaging. However, the group may be too small to conclude and needs to be confirmed in the next studies.

To sum up, in everyday practice, ophthalmologists need to decide which NTG patients need neuroimaging in order to distinguish between cupping caused by glaucoma and potentially life-threatening conditions. According to the results of this study, compressive neuropathy is a rare cause underlying disc excavation and the decision to perform neuroimaging should be made after a detailed assessment of clinical status, rather in the event of finding the signs of possible compressive optic neuropathy than as an obligation. The unilaterality of the disease should remain as an important indication for neuroimaging in NTG patients, although underlying compressive neuropathy is rare. Our experience shows that a diagnosis of compressive optic neuropathy does not exclude NTG. Additionally, some pathologies observed in MRI scans seem to be a rather accidental finding not affecting the visual pathway, which should always be taken into consideration.

## Figures and Tables

**Table 1:** Acquisition parameters of the brain and orbit examination.

	TR	TE	TI	ETL	Slice thickness	Slice spacing	FOV	NEX	Matrix size
	(ms)	(ms)	(ms)	—	(mm)	(mm)	(cm).	—	—
Ax T1 fat sat^∗^	499	10.4	—	4	3.0	0.3	16	3	256 × 192
Ax T2 fat sat	2742	86	—	16	3.0	0.3	16	4	288 × 224
Ax T1	394	10.4	—	4	3.0	0.3	16	3	256 × 192
Cor STIR	6352	30	150	13	3.0	0.5	16	2	320 × 224
Cor T1 fat sat^∗^	559	12.2	—	4	3.0	0.5	16	2	320 × 224

∗Sequences with the same parameters were acquired after intravenous administration of paramagnetic contrast agent.

**Table 2:** The demographic and clinical characteristics of the studied group.

Feature	Studied group
Number of patients	126
Age	66.7 years ± 11.6years
Gender	78 F/48 M
Unilateral glaucoma	72 patients
Laterality of unilateral glaucoma	38 right/29 left
Baseline IOP	17.6 mmHg ± 2.6 mmHg
C : D ratio	0.78 ± 0.12
Central corneal thickness	545.5 *μ*m ± 35.7 *μ*m
MD	−8.61 dB ± 13.4 dB
RNFL thickness	68.9 *μ*m ± 31.2 *μ*m
Peripapillary atrophy	63 eyes (35%)

**Table 3:** Number of patients diagnosed because of additional reasons.

	Reason for neuroimaging	Number of patients (% of study group)	Clinically significant result (% of patients examined for this reason)	Statistical analysis ANOVA with Kruskal-Wallis test as a post hoc
1	Unilateral normal-tension glaucoma	72 (57.1%)	5 (6.9%)	*P* < 0.0001 1 vs. 7, *P* = 0.0002 5 vs. 7, *P* = 0.0055 6 vs.7, *P* = 0.0002
2	The damage in the visual field inconsistent with optic disc appearance	9 (7.1%)	2 (22.2%)	
3	Fast visual field progression (1.5 dB per year or more) assessed according to at least 3 reliable visual field results	5 (4.0%)	2 (40%)	
4	Worsening of visual acuity of at least 2 lines in the Snellen chart connected neither with lens nor retinal pathology	4 (3.1%)	2 (50%)	
5	Optic disc excavation accompanied by pallor	11 (8.7%)	2 (18.1%)	
6	Patients diagnosed under the age of 50	23 (18.2%)	0 (0%)	
7	Scotoma restricted by a vertical line	7 (5.5%)	5 (71.4%)	

## Data Availability

The data used to support the findings of this study are available from the corresponding author upon request.
